# Assessment of antimicrobial stewardship programmes and antibiotic use among children admitted to two hospitals in Sierra Leone: a cross-sectional study

**DOI:** 10.1186/s13756-024-01425-1

**Published:** 2024-07-22

**Authors:** Ibrahim Franklyn Kamara, Bobson Derrick Fofanah, Innocent Nuwagira, Kadijatu Nabie Kamara, Sia Morenike Tengbe, Onome Abiri, Rugiatu Z. Kamara, Sulaiman Lakoh, Lynda Farma, Abibatu Kollia Kamara, Binyam Hailu, Djossaya Dove, James Sylvester Squire, Selassi A. D’Almeida, Bockarie Sheriff, Ayeshatu Mustapha, Najima Bawa, Hailemariam Lagesse, Aminata Tigiedankay Koroma, Joseph Sam Kanu

**Affiliations:** 1 Reproductive Maternal Newborn Child and Adolescent Unit, Universal Health Coverage, Life Course Cluster, World Health Organization Country Office, 21 A-B Riverside Off Kingharman Road, Freetown, Sierra Leone; 2 Ministry of Health, Fourth Floor, Youyi Building, Brookfields, Freetown, Sierra Leone; 3 Pharmacy Board Sierra Leone, New England Ville, Freetown, Sierra Leone; 4 College of Medicine and Allied Health Sciences, University of Sierra Leone, Freetown, Sierra Leone; 5 United States Centers for Disease Control and Prevention Country Office, Emergency Operation Centre, Wilkinson Road, Freetown, Sierra Leone; 6 Department of Demographic and Social Statistics, National Institute of Statistics, Yaoundé, Cameroon; 7 Momentum Country and Global Leadership, Sir Samuel Lewis Road, Freetown, Sierra Leone; 8 United Nation Children’s Fund, Jomo Kenyatta Road, New England Ville, Freetown, Sierra Leone; 9 Reproductive maternal newborn child and adolescent health unit, Universal Health Coverage, Life Course Cluster, WHO Country Office in Sierra Leone, Freetown, Sierra Leone

**Keywords:** Antimicrobial stewardship programmes, Antibiotics use, Children, Point prevalence survey, Sierra Leone

## Abstract

**Introduction:**

Antimicrobial resistance (AMR) is a global public health concern and irrational use of antibiotics in hospitals is a key driver of AMR. Even though it is not preventable, antimicrobial stewardship (AMS) programmes will reduce or slow it down. Research evidence from Sierra Leone has demonstrated the high use of antibiotics in hospitals, but no study has assessed hospital AMS programmes and antibiotic use specifically among children. We conducted the first-ever study to assess the AMS programmes and antibiotics use in two tertiary hospitals in Sierra Leone.

**Methods:**

This was a hospital-based cross-sectional survey using the World Health Organization (WHO) point prevalence survey (PPS) methodology. Data was collected from the medical records of eligible patients at the Ola During Children’s Hospital (ODCH) and Makeni Regional Hospital (MRH) using the WHO PPS hospital questionnaire; and required data collection forms. The prescribed antibiotics were classified according to the WHO Access, Watch, and Reserve (AWaRe) classification. Ethics approval was obtained from the Sierra Leone Ethics and Scientific Review Committee. Statistical analysis was conducted using the SPSS version 22.

**Results:**

Both ODCH and MRH did not have the required AMS infrastructure; policy and practice; and monitoring and feedback mechanisms to ensure rational antibiotic prescribing. Of the 150 patients included in the survey, 116 (77.3%) were admitted at ODCH and 34 (22.7%) to MRH, 77 (51.3%) were males and 73 (48.7%) were females. The mean age was 2 years (SD=3.5). The overall prevalence of antibiotic use was 84.7% (95% CI: 77.9% – 90.0%) and 77 (83.8%) of the children aged less than one year received an antibiotic. The proportion of males that received antibiotics was higher than that of females. Most (58, 47.2 %) of the patients received at least two antibiotics. The top five antibiotics prescribed were gentamycin (100, 27.4%), ceftriaxone (76, 20.3%), ampicillin (71, 19.5%), metronidazole (44, 12.1%), and cefotaxime (31, 8.5%). Community-acquired infections were the primary diagnoses for antibiotic prescription.

**Conclusion:**

The non-existence of AMS programmes might have contributed to the high use of antibiotics at ODCH and MRH. This has the potential to increase antibiotic selection pressure and in turn the AMR burden in the country. There is need to establish hospital AMS teams and train health workers on the rational use of antibiotics.

## Introduction

Antimicrobial resistance (AMR) is a global public health concern impacting individuals throughout their life course [1, 2]. Microorganisms adapt and acquire the ability to proliferate in the presence of previously effective antimicrobial agents, posing a significant challenge to the treatment of infectious diseases [3, 4]. The recent global estimate on AMR mortality states that there were 5 million deaths associated with AMR in 2019. One in five of these deaths occurred among children under the age of five [5]. In the same year, an estimated 2,200 deaths were directly attributed to AMR, and another 9,700 deaths were associated with AMR in Sierra Leone. . AMR-related deaths in Sierra Leone were higher than those from , cardiovascular diseases (9000), maternal and neonatal disorders (8000), and neoplasms and other non-communicable diseases (7500) [6].

AMR disproportionately affects women and children, thereby exerting a detrimental impact on human capital development and child survival [7–10]. Children, especially neonates whose immune systems are immature, and those living in squalor are particularly susceptible to resistant infections [11–13]. Excessive exposure to antibiotic in children is associated with an increased risk of allergies, atopic dermatitis, obesity, autism spectrum disorders, neurodevelopment disorders, destruction of microbiota, and AMR [14, 15]. The effect of AMR on children is further compounded by the limited paediatric-specific formulation antibiotic clinical trials to introduce new effective treatment options [16].

AMR is a natural phenomenon, however, the escalating global burden is attributable to inadequate infection prevention and control (IPC) measures, weak laboratory and surveillance systems, and the inappropriate use of antimicrobial agents especially antibiotics in hospitals [17–19]. One of the proven interventions to curb the rising AMR burden is the promotion of rational antibiotic use by establishing antimicrobial stewardship (AMS) programmes in hospitals. Evidence from countries with hospital AMS programmes indicate that judicious use of antibiotic can optimize patient outcomes, minimize the risk of adverse effects, promote the cost-effectiveness of treatment, and mitigate levels of AMR [20–24].

A nationwide point prevalence survey (PPS) conducted in 2021 in Sierra Leone documented high usage (73.7%) of antibiotics among patients admitted to 26 hospitals. The prevalence was highest in the paediatric wards [25]. Additionally, a study conducted in a regional referral hospital in Sierra Leone among 777 children less than 2 years old revealed that all the children received an antibiotic for acute respiratory tract infections diagnosed based on the Emergency Triage Assessment and Treatment (ETAT) protocol [26]. To date, only Connaught hospital which provides mainly medical and surgical healthcare services to adults has an established AMS programme [27]. However, some secondary and tertiary hospitals in the country have drug and therapeutic committees (DTCs) that should ensure the rational prescribing of antibiotics to patients.Furthermore, although a national PPS reported high usage of antibiotics, research evidence from the assessment of AMS infrastructure, policy and practice, and monitoring mechanism and the prevalence of antibiotics use among children in Sierra Leone is scarce. Given these evidence gaps, the increased risk of AMR, and the adverse long-term health outcomes of high antibiotic use among children, we conducted a study in two hospitals in Sierra Leone that were excluded in the 2021 national PPS to describe the hospitals’ AMS structures, determine the prevalence of antibiotic use among inpatient children, classify the prescribed antibiotics according to the World Health Organization (WHO) Access, Watch, and Reserve (AWaRe) antibiotic categorization and examine factors associated with antibiotic use. The findings from this study will add to the national and global body of evidence on AMS structure and antibiotic use among admitted paediatric patients.

## Methods

### Study design

This was a hospital-based cross-sectional survey using the WHO PPS methodology [28]. Th PPS methodology provides a step-by-step approach for a point prevalence approach to determine antibiotic use among admitted patients in acute care hospital settings. It gives a snapshot of antibiotic use in hospitals at a given time point.

### Study general setting

Sierra Leone is a country in West Africa with a population of about 8 million [29]. The country is struggling with a double burden of communicable and non-communicable diseases (NCDs). However, the burden of communicable, maternal, neonatal, and nutritional diseases is slightly greater than NCDs [30]. It has a high under-five mortality rate with 105 deaths per 1000 live births reported in 2021 [31]. The health care system is structured in three tiers: primary, secondary, and tertiary. The secondary and tertiary hospitals provide both outpatient and inpatient services to children and adults. Ola During Children’s Hospital (ODCH) and Makeni Regional Hospital (MRH) are both tertiary hospitals as stated in the national essential health services package [32].

## Study specific settings

### Ola During Children’s Hospital (ODCH)

The ODCH is a government-owned, national referral paediatric hospital located in the capital city of Freetown [33]. The hospital has a bed capacity of 164. It comprises of a high dependence unit, a intensive care unit, a step-down (transition ward), a therapeutic feeding unit, a Special Care Baby Unit (SCBU) or neonatal unit, an infectious disease unit, a Diarrhea Treatment and Training Unit (DTTU), and a cancer unit.

### Makeni Regional Hospital (MRH)

The MRH is a government-owned facility and is located in Bombali district, Northern Sierra Leone. It is located 170 km away from Freetown, the capital city of Sierra Leone [34]. It is a 200-bed referral centre for the northern province, with 50 beds for paediatric services. It has a paediatric ward, an inpatient feeding centre, and an SCBU. The hospital is also a site for the Child Health and Mortality Prevention Surveillance (CHAMPS) project that is being implemented in Bangladesh, Ethiopia, Kenya, Mali, Mozambique, Sierra Leone, and South Africa to investigate the causes of death including pathogens through bacteriology [35].

### Study period

In both hospitals, data was collected between 6^th^ and 17^th^ June 2022 and lasted for 3-5 days per hospital.

### Hospital sampling

According to the WHO PPS methodology [28], we selected these two hospitals using a convenience sampling approach [28]. ODCH and MRH were selected as both hospitals were not included in the nationwide PPS on antibiotic use conducted in 2021. Additionally, these hospitals are tertiary hospitals and provide paediatrics inpatient healthcare services. Furthermore, they are part of the Fleming Fund project to support the strengthening of laboratory capacities (bacteriology) and AMS initiatives.

## Inclusion and exclusion criteria for wards, patients, and antibiotic administration

### Inclusion criteria

Only paediatric inpatient wards were included. In each hospital, records of patients aged 17 years old or below, and who were hospitalized in the ward before or at 08:00 am on the day of the survey were considered eligible. Records of all neonates born before 08:00 am on the day of the survey were also eligible. Antibiotics administered through parenteral, oral, sublingual, and inhalation routes were included. Records of all patients meeting the eligibility criteria were included in the survey irrespective of whether they received antibiotic treatment or not [28].

### Exclusion criteria

Records of paediatric patients seen in the outpatient departments and emergency rooms were excluded from the survey. The records of patients admitted to the ward after 08:00 am, patients whose antibiotic therapy was initiated after 08:00 am on the day of the survey, and patients whose antibiotic therapy was stopped before 08:00 am on the day of the survey were also excluded. Antibiotics administered through drops and other topical applications were also excluded [28].

### Data collection

Data was collected using the WHO PPS methodology hospital questionnaire and, the required PPS data collection forms. The hospital questionnaire has several indicators under three headings: infrastructure; policy and practice; and monitoring and feedback. Each of the questions required either a yes or no response or an absolute count [28]. The PPS forms collect basic information from medical records and associated patient documentation which are relevant for treatment and management from all the eligible patients. Information on AMS capacities, patients, indications, antibiotics, and microbiology were collected.

The research team members (doctors and nurses) were trained on the WHO PPS methodology. The data collection forms were pretested in a similar hospital setting to ensure uniformity during the survey period as part of the data quality assurance mechanism. There was no interaction between patients and the research team, and the process of data collection did not interrupt the provision of healthcare services. Patient identifiers were not documented and a unique code was assigned to each patient record to ensure anonymity. Infections were considered as community-acquired infections (CAIs) if symptoms of infection were present on admission; as healthcare-associated infections (HAIs) if symptoms appeared 48 hours or more after admission; surgical prophylaxis (SP) included any antimicrobial administered to prevent surgical-site infections; and medical prophylaxis (MP) as the use of antibiotics to prevent infections in patients with non-surgical conditions.

### Data analysis

**Fig. 1:**
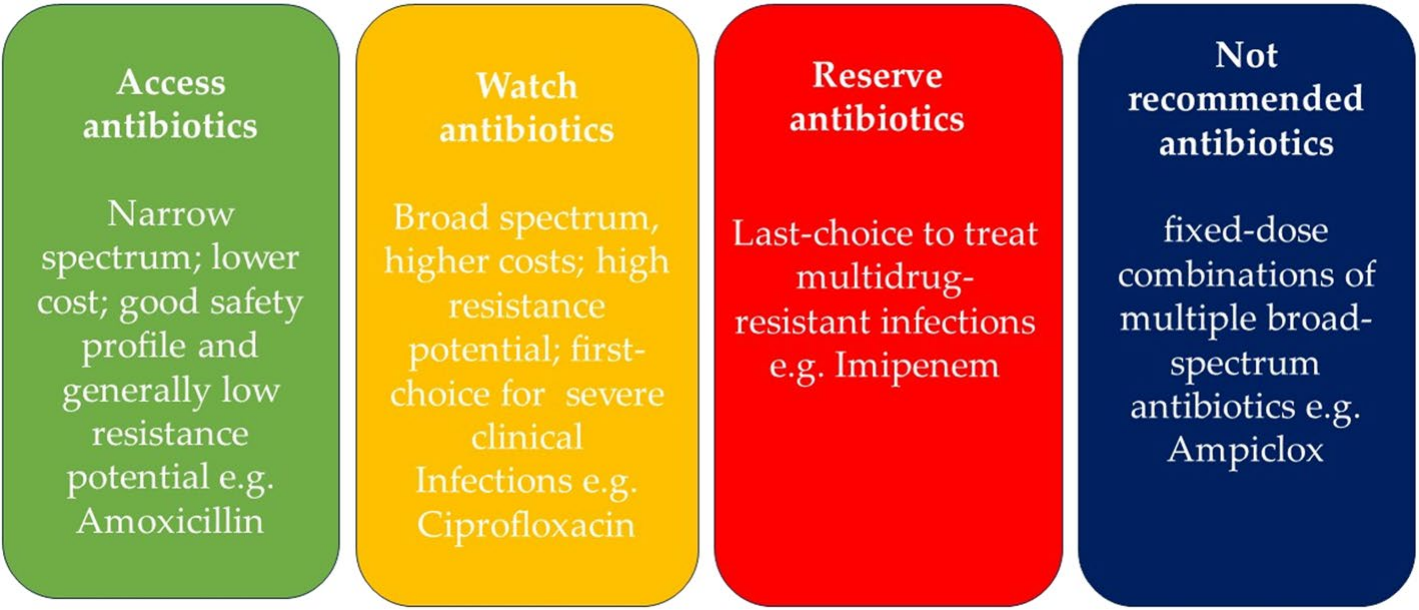
WHO AWaRe antibiotic categorization [36]

Data analysis was performed using SPSS version 22. Descriptive statistical measures such as frequency, proportion, mean, and median were computed to summarize the data. The prevalence of systemic antibiotic use was defined as a percentage of the total number of paediatric patients on any systemic antibiotic at the time of the study against the number of patients on admission. Additionally, the top 5 prescribed antibiotics were determined based on their proportionate consumption and categorized according to the WHO AWaRe categorization (Fig. 1).

The Chi-Square test was used to check for an association between antibiotic use (dependent categorical variable), and the independent categorical variables age group and sex. The Chi-Square test was used to assess the relationship between the independent variables on the dependent variable, and a backward stepwise binary logistic regression was then performed to determine the explanatory factors of antibiotic use. Missing data was handled via multiple imputations in the regression analysis. A *p*-value of <0.05 was considered statistically significant.

## Results

### Antimicrobial stewardship structures

AMS structures were non-existent at ODCH and MRH to support the rational use of antibiotics. Only 4 out of the 15 infrastructural components were present in ODCH and 1 in MRH. The components that are present at ODCH are functional Drugs and Therapeutics (DTC), Infection Prevention and Control (IPC) Committees, Information communication and technology, and a pharmacist responsible for appropriate prescribing. MRH has access to the CHAMPS laboratory for research purposes. No component in terms of policy and practices and monitoring and feedback were present in both hospitals (Table 1).

**Table 1:**
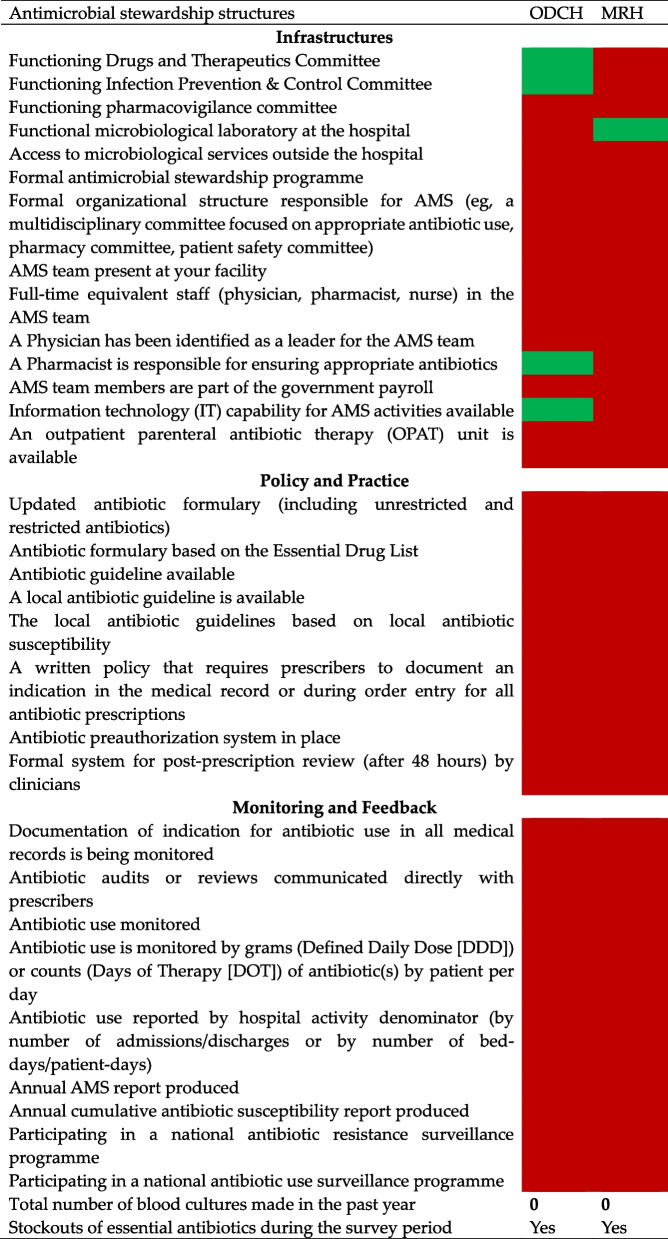
Antimicrobial stewardship structures at ODCH and MRH

*ODCH* Ola During Children’s Hospital, *MRH *Makeni Regional Hospital,  Yes  No

### Demographic characteristics of the study participants at ODCH and MRH

Of the 150 paediatric patients included in this survey, 116 (77.3%) were admitted at ODCH and 34 (22.7%) at MRH; 77 (51.3%) were aged less than 1 year, and 77 (51.3%) were males whereas 73 (48.7%) were females. The mean age was 2 years (SD=3.5) (Table 2). All patients were admitted for less than 30 days, and there were no intubations, transfers from other hospitals, or surgeries performed during the period of admission.

**Table 2:** Characteristics of the study participants at ODCH and MRH

Characteristics	Frequency (n)	Percentage (%)
Hospital		
ODCH	116	**77.3**
MRH	34	22.7
Sex		
Male	77	**51.3**
Female	73	48.7
Age Group		
<1 year	77	**51.3**
1-4 years	49	32.7
5-17 years	21	14.0
Missing	3	2.0
Peripheral vascular catheter		
Yes	139	**92.7**
No	11	7.3
Place of Admission		
Non-ICU	142	**92.0**
ICU	12	8.0
Mean age (years), (SD)	1.99 (3.5)	

*ODCH* Ola during children's hospital, *MRH* Makeni regional hospital, *ICU* Intensive Care Unit, *SD* Standard deviation

### Prevalence and indications for antibiotic use, and blood culture requests

The overall prevalence of antibiotic use was 84.7% (95% *CI*: 77.9%-90.0%). Over two-thirds (68, 88.3%) of the children aged less than one year received antibiotics. Relatively, more males (68, 88.3%) were given antibiotics compared to females (59, 80.8%) (Table 3). CAIs were the only diagnoses for the initiation of antibiotic treatment. HAIs, SP, and MP were not indications for antibiotic use. Blood culture was not requested for any of the patients before the initiation of prescribed antibiotic treatment.

**Table 3:** Prevalence of antibiotic use by hospital, age group, and sex

Variables	Frequency (n)	Prevalence (%)	95% CI
Overall	127	**84.7**	77.9-90.0
Hospital			
MRH	34	**100**	
ODCH	93	80.2	72.1-87.2
Age Group			
< 1 year	68	**88.3**	81-95.7
1-4 years	39	79.6	67.9-91.3
5-17 years	17	81.0	62.6-99.3
Sex			
Male	68	**88.3**	80.5-95.5
Female	59	80.8	71.2-89.9
Average number of prescribed antibiotics- **2.4**			
WHO recommended outpatient antibiotic prescription per patient-1.6-1.8			

*ODCH* Ola during children's hospital, *MRH* Makeni Regional hospital, *WHO* World Health Organization

A total of 364 prescriptions of antibiotics were included in the study for 127 paediatric patients. Nearly all (353, 97%) of the prescribed antibiotics were administered through the intravenous (IV) route. Approximately half of the patients received 2 antibiotics (58, 47.2%); the lowest number of antibiotics prescribed was 1, the highest was 6 and the mean was 2.4. There were a few (13,5, 6.3%) missed doses of the prescribed antibiotics.

**Fig. 2:**
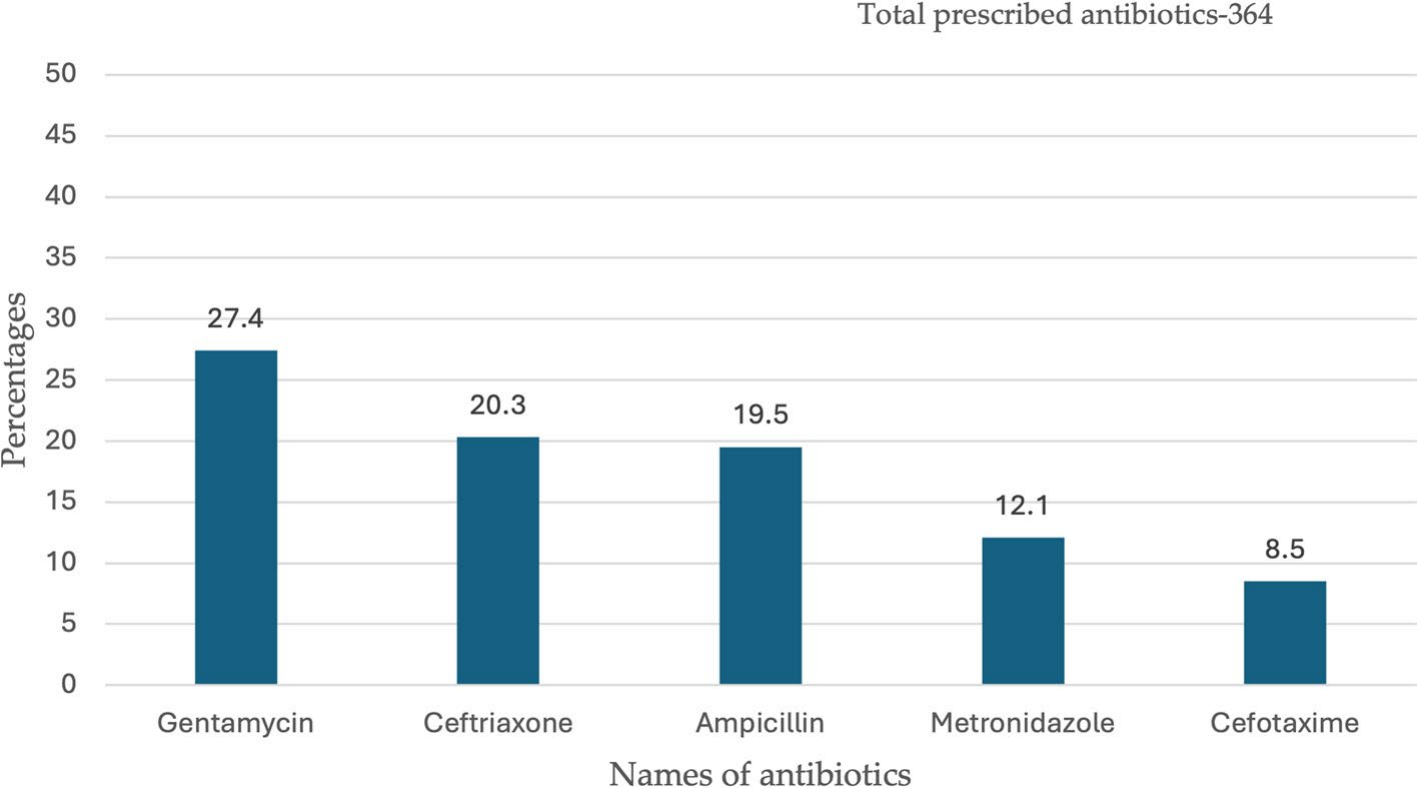
Top five antibiotics prescribed to patients admitted at ODCH and MRH

The top five antibiotics prescribed for patients were gentamycin-Access (100, 27.4%), ceftriaxone-Watch (76, 20.3%), ampicillin-Access (71, 19.5%), metronidazole-Access (44,12.1%) and cefotaxime-Watch (31, 8.5%) (Fig. 2). The majority (152.3, 77.2%) of antibiotics were written in International Non-proprietary Names (INN)

**Fig. 3:**
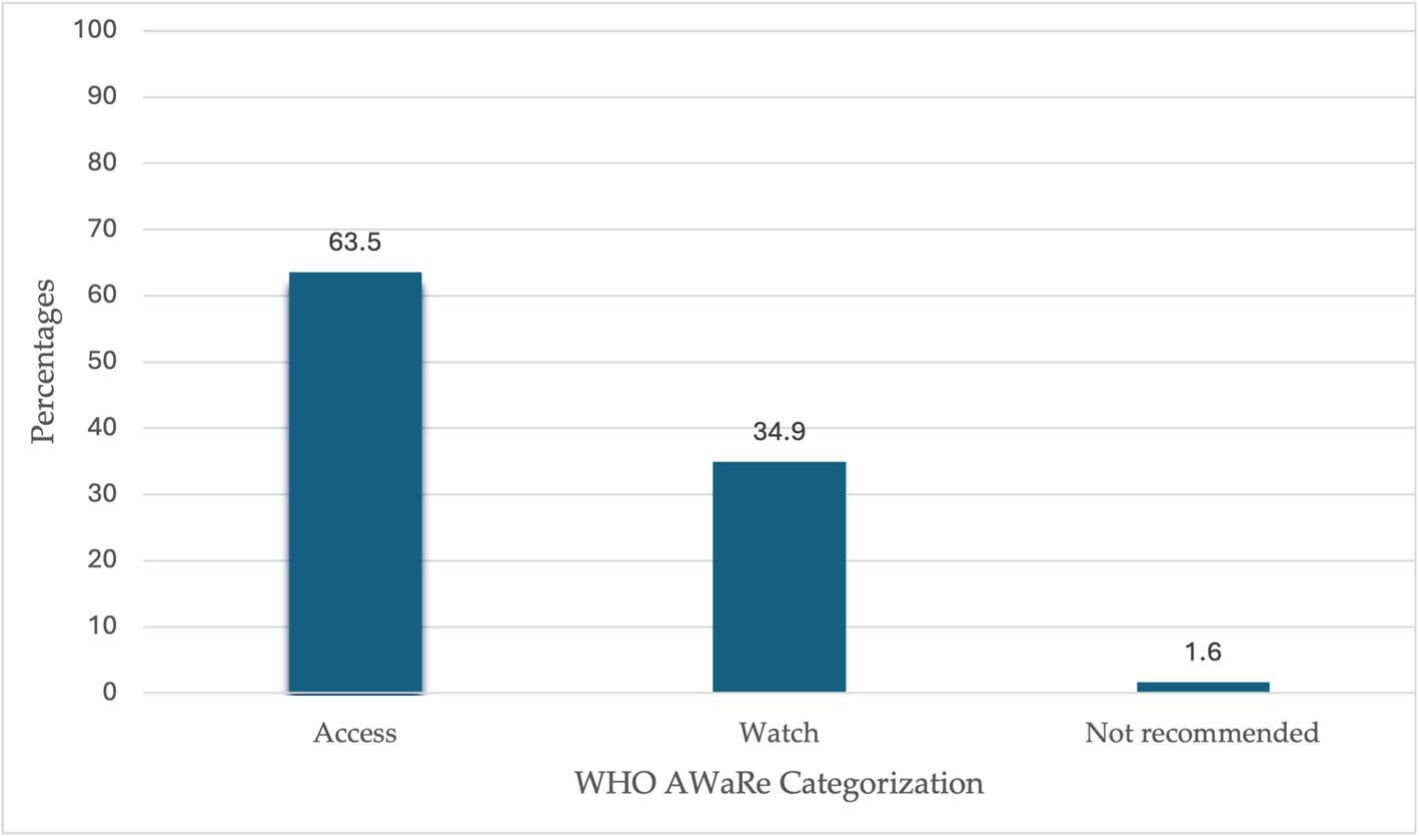
Percentage distribution of antibiotics prescribed to patients according to the WHO AWaRe Categorization

The larger proportion (231, 63.5%) of the antibiotics prescribed fell in the access group of antibiotics according to the WHO AWaRe categorization. No reserve antibiotic was prescribed to the patients (Fig. 3).

## Factors associated with antibiotic use

The findings from the logistics regression showed that sex and age group had no association with antibiotic use (Table 4).

**Table 4:** Factors associated with antibiotics use

Variable	Category	AOR	95% CI	*P*-value
Age group	<1 year	1.80	0.49-6.62	0.38
	1-4years	0.85	0.23-3.14	0.81
	5-17 years	Reference		
Sex	Male	1.77	0.71-4.39	0.22
	Female	Reference		

*AOR* Adjusted odd ratios

## Discussion

To our knowledge, this is the first study to assess AMS capacities in hospitals in Sierra Leone using the WHO PPS methods. Additionally, no prior study has focused only on assessing inpatient antibiotic use among children using this methodology.

Neither ODCH nor MRH had an established AMS programmes. Therefore, there is a weak oversight structure to ensure rational use of antibiotics among children admitted to these hospitals. In contrast to our findings, a study conducted in Thailand that assessed the establishment of AMS programmes and antibiotic use in 41 hospitals documented that nearly all of the hospitals have functional antibiotic stewardship programmes to guide rational prescribing [37]. We found that only 4 out of the 15 infrastructural components were present in ODCH and 1 in MRH. No component in terms of policy and practices and, monitoring and feedback were present in both hospitals. Additionally, there was high usage of antibiotics among the admitted children. ODCH had functional IPC and DTC committees and a pharmacist responsible for antibiotic prescribing. However, there was no AMS team, AMS committees, antibiotic formularies, antibiotic guidelines, or other written policies to provide guidance on indications for antibiotic use in both hospitals. Furthermore, antibiotic use and consumption were not monitored in both hospitals and yet this is a requirement for participation in the national antibiotic resistance and use surveillance program. However, since these hospitals are benefiting from the Fleming Fund project that is geared towards strengthening bacteriology capacities to do culture and sensitivity, this project will support any AMS intervention that will be initiated in these hospitals. This is a low-hanging fruit as the renovation and equipping of their laboratories are close to completion and will be functional before the end of the year 2024.

The lack of AMS programmes in these hospitals may have contributed to the high usage of antibiotics (84.7%). We consider this prevalence of antibiotic use to be high as over 60% of paediatric admissions in Sierra Leone are a result of malaria infections [38]. Our findings are similar to the prevalence of antibiotic use (85.7%) among the paediatric population in the national PPS conducted in 2021. The high usage of antibiotics in these two hospitals is likely to be replicated in other hospitals in the country except Connaught Hospital that has an established AMS programme [27] and recorded a lower prevalence (67%) in the national PPS as compared to other hospitals. Additionally, the over-reliance on algorithm systems like ETAT in paediatric healthcare may also be a contributing factor. The syndromic pattern of these algorithms likely influences on the early initiation of antibiotics. Furthermore, the competencies of healthcare workers to implement these algorithms might also play a part in the high usage of antibiotics. Routine capacity building, mentorship, and coaching might promote the appropriate use of algorithms like ETAT. The high usage of antibiotics among these admitted children has the potential to impact negatively the quality of care being provided; this is because they will potentially be exposed to medication toxicity such as unfavourable side effects and allergic reactions. There is an urgent need to establish AMS programmes in these hospitals to guide rational antibiotic prescribing. Studies done in South Africa, Kenya, Sudan, Tanzania, and Egypt have all shown that AMS programmes are effective in optimizing the use of antibiotics in hospitals [39]. Moreover, there is a need for monitoring the implementation of the national ETAT protocol and other treatment algorithms in the country; and conducting refresher training for healthcare workers to ensure rational prescribing of antibiotics for the paediatric patients who are at increased risk of suffering due to AMR and its consequences.

We observed that the majority (85%) of the children admitted to ODCH and MRH received antibiotics. This prevalence, even though limited to the paediatric population, exceeds the prevalence in the Sierra Loene’s national PPS, the study on antibiotic use among admitted COVID-19 patients [3, 25], and an outpatient antibiotic use study conducted in Sierra Leone [40]. In concordance with our findings, in the national PPS, antibiotic use was highest in the paediatric wards (85.7%) [25]. On the contrary, a study conducted in Thailand which included records from 41 hospitals documented a lower prevalence of antibiotic use among neonates (32.5%) and children (32.5%) [37]. Another study conducted in 16 general and children’s hospitals in China documented a lower (66.1%) prevalence [41]. The overuse of antibiotics is not only common in Sierra Leone but also in other parts of the world. A recent narrative review showed that about 50% of children with viral infections who accessed care at hospitals in Europe received antibiotics [42]. Presently, there is no inpatient antibiotic use threshold, however, the WHO recommends that the level of outpatient antibiotic use should be approximately 30%; additionally to this recommendation, about 50% of antibiotics prescribed to patients are considered inappropriate [36]. In our study, antibiotic use was highest in children less than one year. It is possible that these infants were given antibiotics on the backdrop that their vulnerability to bacterial infections was high during the oral phase of the development. Furthermore, clinicians might also assume that they are prone to infections as their immunity is not well developed as compared to adolescents and adults. Moreover, neonates were given antibiotics for non-infectious medical conditions like birth asphyxia. Clinicians believe that birth asphyxia is often caused by prolonged labour which predisposes the neonates to aspiration or swallowing of meconium that might lead to bacterial pneumonia. The clinicians initiate antibiotic treatment in a bid to prevent secondary bacterial infections. Likewise, clinicians may initiate antibiotic therapy early among neonates and infants because of their increased predisposition to severe bacterial infections and death; this is presumed to be a safety net within the context of limited access to quality diagnostics (culture and sensitivity) and weak IPC measures in these hospitals.

Antibiotic use was higher in males than in females. These gender variations in antibiotic prescriptions have been documented by studies conducted outside of Sierra Leone [43]. Therefore, it is important to disaggregate AMR and AMU datasets based on gender or sex. It has been shown that women are 27 times more likely to receive an antibiotic in their lifetime as compared to men [44]. Based on our study evidence, we cannot ascertain the underlying reason for males being more likely to receive antibiotic prescriptions than females. A study with a larger sample size may provide evidence on the association between antibiotic use and sex or gender. Additionally, qualitative research should be conducted to explore why the healthcare workers were inclined to prescribe antibiotics to boys as compared to girls.

A higher proportion of patients received two antibiotics – gentamycin and ceftriaxone. The average number of antibiotics prescribed per patient was 2.4. This is higher than the WHO outpatient recommendation of 1.6 -1.8 antibiotics prescribed per patient [45]. A study conducted in Zambia also found an average of 2.5 antibiotics prescribed per patient, which is similar to our study [46]. The top five antibiotics prescribed were gentamycin, ceftriaxone, ampicillin, metronidazole, and cefotaxime. The use of cefotaxime was mainly at the SCBU for neonates. Comparable findings were also documented in a study conducted in China where third-generation cephalosporin was frequently used among neonates [41]. Two of the top five antibiotics in our study were part of the ‘Watch’ group of antibiotics according to the WHO AWaRe categorization. This is different from the national PPS wherein only one ‘Watch’ antibiotic was part of the top five prescribed antibiotics [25]. Most (63.5%) of the antibiotics prescribed fall in the ‘Access’ group of antibiotics. This is in keeping with the WHO guidelines which recommend that at least 60% of antibiotics used in hospitals should be from the ‘Access’ group as they are less toxic and not prone to resistance [47]. This finding is also similar to the evidence on antibiotic use from studies done in Liberia and Ghana [48, 49]. When antibiotic stewardship programmes are established at ODCH and MRH the use of cephalosporins should be controlled with interventions like pre-authorization.

To promote appropriate use of antibiotics at the study hospitals, we recommend the establishment of AMS programmes as a long-term measure [48, 50]. The national AMR coordinating unit should provide technical and operational support to these hospitals to establish AMS programmes. These should factor in critical components such as microbiology capacity, competent AMS personnel, updated essential medicines list and antibiotic formulary, and other policy and practice parameters are out of their control. These hospitals could embark on ‘low-hanging fruits’ persuasive AMS interventions like the establishment of AMS committees, leveraging on the already existing DTC and IPC committees; and go a step further to institute an antibiotic pre-authorization system. In tandem, the scope of the IPC team responsibilities can be expanded to cover AMS initiatives such as training and mentorship, advocacy for adherence to standard treatment guidelines, and sensitization about the paediatric essential medicines list, the WHO AWaRe antibiotics handbook, and monitoring of antibiotic use and resistance trends [51]. Furthermore, a facility-based physician and pharmacist should be appointed to champion AMS activities. We believe that the improvement in microbiology laboratory capacity and the implementation of cost-effective AMS initiatives will promote rational prescribing of antibiotics which will in turn support quality healthcare services for children in these tertiary hospitals that are admitting very sick patients Routine audit and feedback sessions on antibiotic use with clinicians might further motivate rational prescribing of antibiotics among paediatric patient population.

Our study has several strengths. First, we used a global standardized tool to collect data. Second, healthcare workers capable of comprehending patients' notes were trained on the WHO PPS methodology to collect the data. This ensured quality data collection. Third, the data collection tools were pretested before data collection at the study hospitals to ensure uniformity in the data collection process. Fourth, we followed the strengthening reporting of observational study (STROBE) guidelines to report our findings.

Despite the strengths highlighted above, our study had some limitations. Firstly, we did not include the optional variables of the WHO PPS methodology like malaria, tuberculosis, and human immunodeficiency virus statuses. Second, our approach only gives a snapshot of antibiotic use, a detailed clinical audit will provide a better picture of the quality of prescribing practices. Third, during the period of data collection, hospital admission was low that resulted in a low sample size, which might affect the power of our study. Lastly, extrapolation of our study findings should be done with caution as it only represents the two hospitals. However, we have made a case for the establishment of hospital AMS programmes to promote rational antibiotic prescribing in paediatric departments and children's hospitals in the country.

## Conclusion

Our study revealed that most of the children admitted at the ODCH and MRH received at least two antibiotics during their period of hospitalization. Furthermore, almost all the required components of the AMS program were in a fragmented form or completely lacking within the study hospitals. This has the potential to increase antibiotic resistance selection pressure and in turn, increase the burden of AMR in the country. We recommend the immediate establishment of AMS teams and committees at ODCH and MRH and phased implementation of the different components of hospital AMS programmes. Additionally, there should be a strong linkage between the AMS team and the clinical and diagnostic units to ensure the synergy between antibiotic and laboratory stewardship programmes.

## Data Availability

Data are available upon reasonable request.

## Abbreviations

AMR: Antimicrobial resistance
AMS: Antimicrobial stewardship
AWaRe: Access, Watch, and Reserve
ETAT: Emergency triage assessment and treatment
DTTU: Diarrhea treatment and training unit
DTC: Drug and Therapeutic Committee
ICU: Intensive care unit
IPC: Infection prevention and control
ODCH: Ola during children’s hospital
MRH: Makeni regional hospital
PPS: Point prevalence survey
SCBU: Special care baby unit
WHO: World health organization
